# Development of a comfort suit-type soft-wearable robot with flexible artificial muscles for walking assistance

**DOI:** 10.1038/s41598-023-32117-2

**Published:** 2023-03-24

**Authors:** Jiaoli Piao, Minseo Kim, Jeesoo Kim, Changhwan Kim, Seunghee Han, Inryeol Back, Je-sung Koh, Sumin Koo

**Affiliations:** 1 Department of Clothing, Konkuk University, Seoul, 05029 Republic of Korea; 2 Department of Clothing and Textiles, Yonsei University, Seoul, 03722 Republic of Korea; 3 Department of Mechanical Engineering, Ajou University, Suwon, 16499 Republic of Korea

**Keywords:** Diseases, Health care

## Abstract

Anchoring components are added to wearable robots to ensure a stable interaction between the suits and the human body and to minimize the displacement of the suits. However, these components can apply pressure to the body and can cause user dissatisfaction, which can decrease willingness to use the suits. Therefore, this study aims to develop a suit-type soft-wearable robot platform for walking assistance by providing comfortable garment pressure to ensure user satisfaction. The first prototype of a wearable robot suit was developed with anchoring components on the shoulders, waist, and thighs based on previous research results. Wear tests were conducted to measure garment pressure depending on posture using pressure sensors, and satisfaction surveys were conducted. The second prototype design was then developed, and performance tests with flexible artificial muscles and a satisfaction survey were conducted. Regarding the first prototype, the participants felt more than normal pressure in the shoulders and relatively less pressure in the thighs and calves. Thus, compared to the first design, the second design ensured a decreased garment pressure and resulted in an improvement of overall user satisfaction. These results can help provide guidance in the development of wearable robots by taking pressure comfort and user satisfaction into consideration.

## Introduction

A wearable robot is a body-worn robotic system that offers high load, high mobility, and posture persistence capabilities through functions such as posture control, situational recognition, and posture-signal generation in response to various environments^1,2^. The global wearable robot-exoskeleton market is expected to grow with a compound annual growth (CAGR) of 43.6%, from $952.5 million in 2022 to $11,995.7 billion by 2029, with North America holding the largest market share and Asia’s market growing rapidly^3^. A wearable robot can assist human movements by driving actuators based on the movement intentions of the wearer^1,2^.

Wearable robots were developed to make human life easier and to perform dangerous tasks efficiently; however, their rigidity and weight limit natural body movements. Furthermore, research has been lacking on wearable robots that take comfort into consideration from the early stages of the development process. This has led to the development of soft and flexible suit-type wearable robots, wherein anchoring methods are utilized to stabilize the wearable robots on the body^4,5^. The anchoring methods can be classified based on the methods and materials used: elastic forces of stretchy fabrics^6^, stitching techniques^7^, webbing straps^8^, and hook and loop fasteners^9^. However, anchoring components can create uncomfortable garment pressure, limit natural movement, and even cause blood circulation disorders and injuries, which can lead to a discontinuance of use of the wearable robots. Therefore, developing wearable robots that take pressure and user satisfaction into consideration is recommended.

Garment pressure is the pressure generated by the contact between the garment and the human body, and it is attributed to the garment’s weight, the tightening or pulling of the garment material, and the deformation of stretchy fabrics to fit the shape of the human body. An appropriate level of garment pressure can provide positive attributes for the human body (e.g., support and recovery), whereas excessive pressure can hinder movement. Furthermore, garment pressure can cause increased venous blood flow, metabolite removal, and muscle oxygenation because of the reduced cross-sectional area of blood vessels^10–13^. It is also necessary to consider the fabric elongation recovery rate and dimensional change rate after washing because these factors can change the garment pressure.

Compression can be categorized as mild (20 mmHg), medium (20–40 mmHg), strong (40–60 mmHg), and very strong (over 60 mmHg)^14,15^. A suitable pressure for comfort is less than 29.4 mmHg, and the discomfort threshold is approximately 44.10–73.50 mmHg^14,16^. If the compression pressure exceeds 50 mmHg, it may cause ambulatory venous hypertension reduction and vein occlusion while walking^14,15^. Therefore, the comfort zone of garment pressure is considered to be between 20 and 30 mmHg.

Although various types of walking-assistive wearable robots are being developed, there is a lack of research on wearable robots that takes garment pressure and user satisfaction into consideration. Therefore, we attempted to develop suit-type soft wearable robot platforms for daily life walking assistance that are comfortable in terms of garment pressure and that can ensure wearer satisfaction. We expect that the results will help in the development of wearable robots that take pressure comfort and user wear satisfaction into account.

## Methods

### Platform design and prototype development

Different fabrics have unique features that can help a soft-wearable robot suit function better while providing comfort to the wearers. Stretchy fabric can provide stretchiness for comfort and can fit various sizes, while a mesh fabric can provide better ventilation; therefore, these fabrics were applied to areas such as the back of the knees that tend to become sweaty. Meanwhile, the nonstretchy fabric can assist in limiting the pulling power to minimize the dislocation of important components such as actuators, anchoring lines and other suitable details.

**Figure 1:**
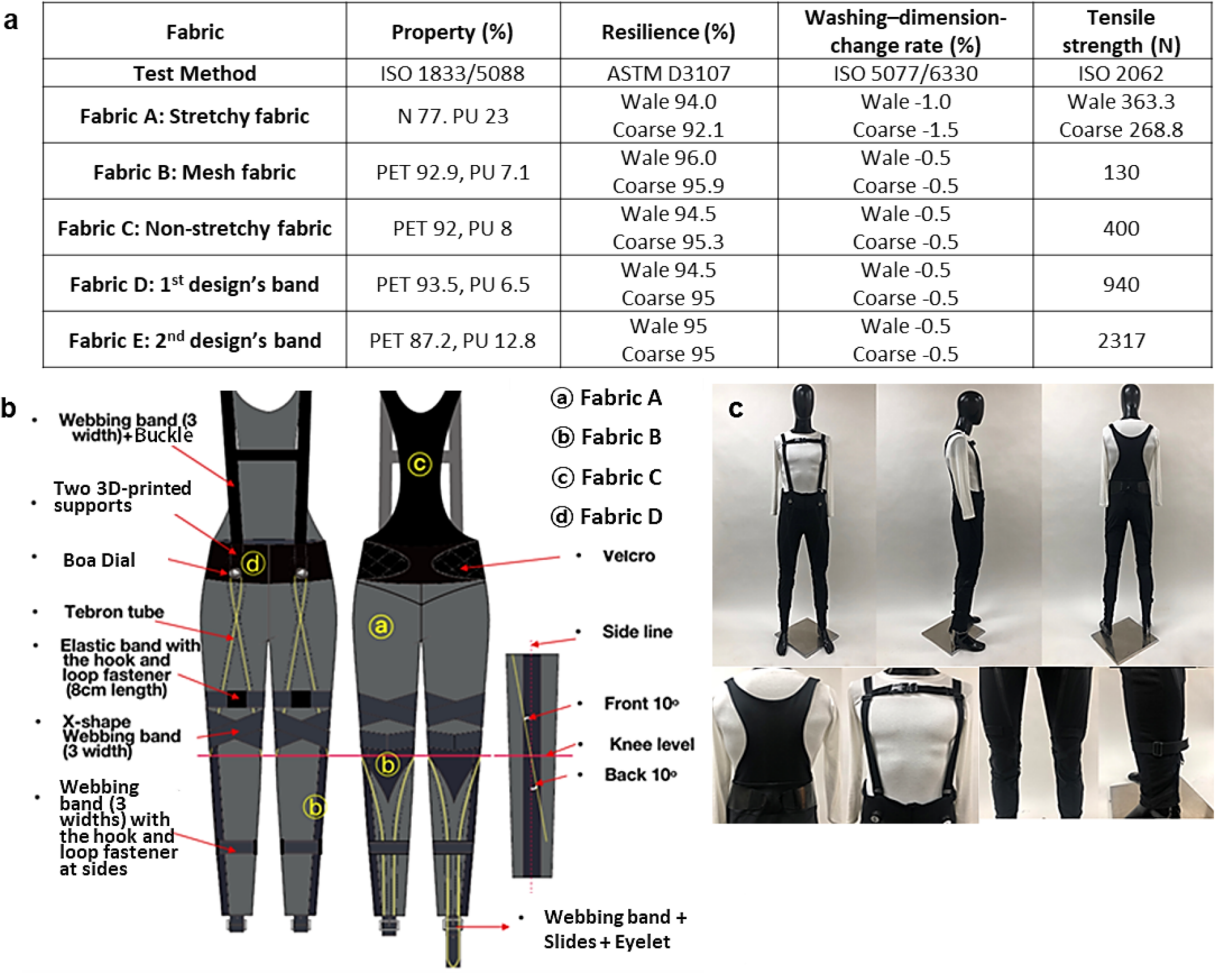
First design. (**a**) Results of performance tests of applied fabrics. (**b**) First design and specifications. (**c**) Developed prototype based on the first design.

**Figure 2:**
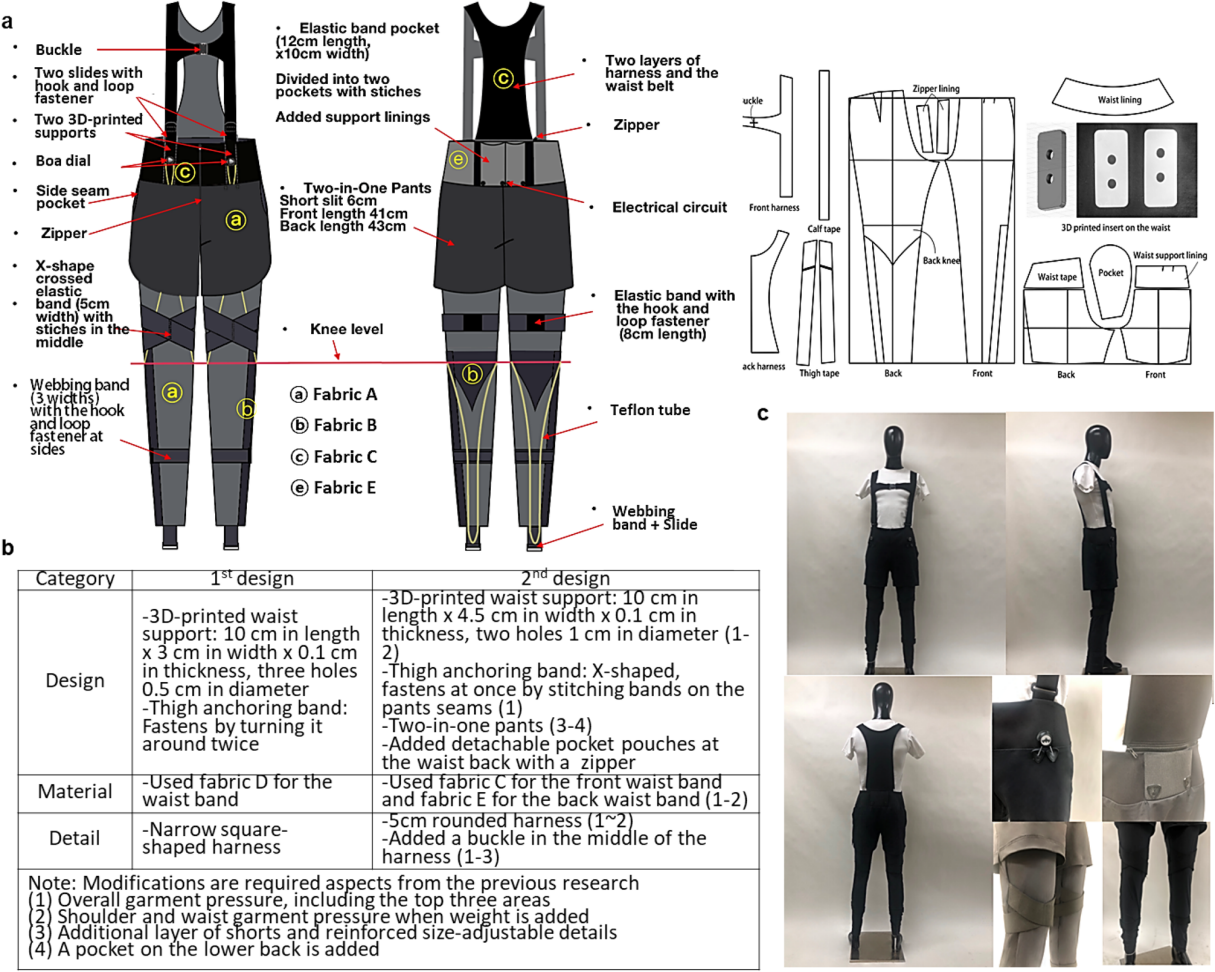
Second design. (**a**) Second design and specification, (**b**) modified aspects of the second design from the first design. (**c**) Developed second prototype.

Compression is achieved by the elongation of fibers and threads, and the elasticity of the material significantly affects the changes in pressure on the body^18^. Therefore, fabrics with excellent elongation-recovery rates and washing-dimension-change rates, which affect garment pressure, were used^19^, as shown in Fig. 1a. Fabric A is a stretchy fabric that has the highest tensile strength (wale 363.3 N, course 268.8 N). Fabric B is a wicking fabric located at the side and back of the pants, which are the areas prone sweatiness, and thus, the fabric can provide comfort. Fabric C is a nonstretchy fabric with high tensile strength; it was used at the waist to provide strong anchoring. Fabric D is 7 mm thick and has a high tensile strength of 256% and 2317 N; therefore, it was used in the first design in the thigh and calf areas, where durability is required for walking. Fabric E is a band material with a sufficient elongation-recovery rate and high tensile strength, and it was used in the second design (Fig. 2a).

Two flexible supports of 10 cm (length) × 3 cm (width) × 0.1 cm (thickness) pieces were printed with thermoplastic polyurethane (TPU) with three 0.5-cm holes using a 3D printer (Shindo 3D WOX 2X). The supports were inserted into the waist belt, and the front harness was connected to the waist belt. The Boa dials were positioned in a straight line to enhance stability when the nitinol wires contracted and pulled at the suit for walking assistance, as shown in Fig. 1b,c. For the routing lines, the linear actuator consisted of nitinol wires with Teflon tubes, which crossed each other above the knees and past the sides of the knees at 10°. The wearers were able to adjust the linear actuator’s length with the Boa dial and the buckles to fit the actuator to their legs, and the harness was detachable.

The first design was modified based on the garment pressure measurement and survey results, and the second design was developed as shown in Fig. 2. The two designs were developed to be size-adjustable for practical use and commercialization. Notably, customizations based on each person’s body size will highly increase the cost. Therefore, to achieve the size-adjustable function, the two prototypes used Boa dials, buckles and slides, hooks and loops, webbing bands, stretchy fabrics, and elastic bands to change the lengths and widths of the suits as desired. The horizontal webbing band and the buckle crossing the chest helped adjust the width of the torso. The waist band could be adjusted using the hooks and loops, and all anchoring parts were adjustable using the slides. Adjustable elastic bands were placed on the thighs, where the large muscles change body size more frequently than in other areas, to anchor the devices while ensuring comfort. The lengths of the routing lines of the actuator could be adjusted with the Boa dial, and the webbing strap on the pants hemlines were length-adjustable with the webbing straps and slides. The stretchy fabric allowed adjustments for the pants overall in terms of the width and the length, and the nonstretchy fabric was used for the back of the torso back to provide strong support.

### Textile-based flexible artificial muscle design for ankle assistance

Shape memory alloys (SMAs) exhibit hysteresis behavior because of a nonlinear thermoelastic material response^20^. SMAs have the drawback of low controllability and inaccurate positioning^21^; however, differences in Young’s modulus of the austenite and martensite phases can provide more comfortability in user movements (i.e., when the SMA is extended while the user swings their legs). Therefore, hysteresis behavior in the SMA would be helpful for both actuating and releasing states. A high modulus in the actuation state provides high force assistance for the ankle, and a low modulus in the releasing state reduces the disturbance in stepping on the ground.

**Figure 3:**
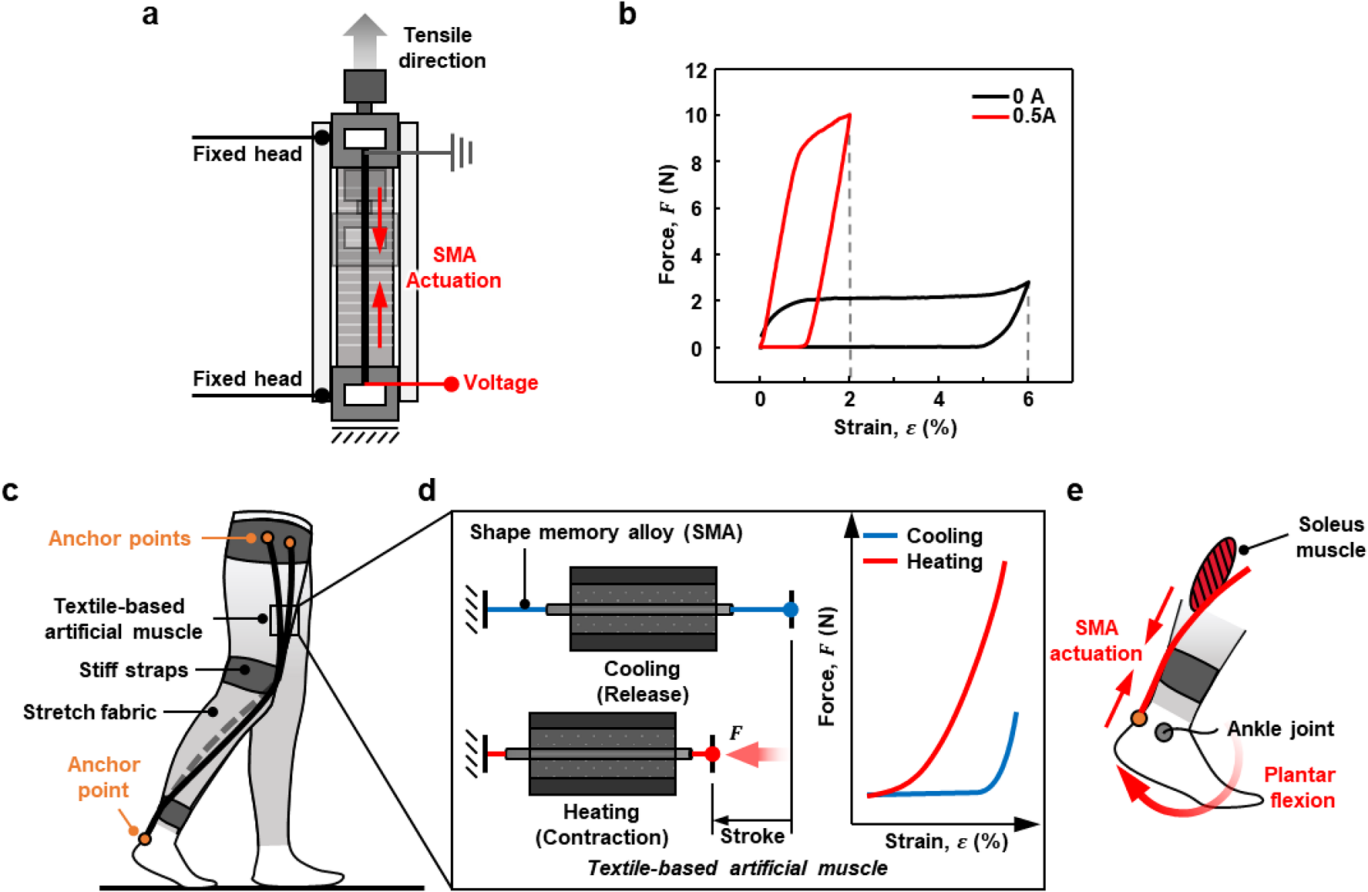
Characterization of the SMA actuator and conceptual actuator design of a suit-type soft wearable robot. (**a**) Schematic diagram of the experimental setup for tensile testing. (**b**) Measured 0.006-mil SMA blocked force as functions of strain and input current. (**c**) Overview of the suit-type soft wearable robot. (**d**) The cooling and heating of the SMA actuators. (**e**) The torque inducing the plantar flexion of the ankle.

The cooling rate of the SMA is determined by thermal diffusion and convection. Many studies have been reported to reduce cooling time using water, fans, and lower diameter SMA^22–24^. We attempted to find the optimal fabrics integrated with SMA to reduce cooling time and adopted a small diameter (0.006 mil) to enable assisted walking (Fig. 3a,b).

We designed a textile-based flexible artificial muscle actuation system to provide parallel assistance to the plantar flexor muscles (soleus muscle) during normal gait, as shown in Fig. 3c. Figure 3c shows routing lines and anchor points on the robot for driving torque at the ankle joint. Webbing straps (gray) are placed and fixed on the waist, knee, and calf joint to effectively transmit the contraction force generated by SMA actuators to the ankle. Each end of the SMA wire is anchored to the webbing strap at body parts that can endure high force and stress.

The SMA actuators require a simple electronics system and have their own unique characteristics of shape memory effects (SMEs)^25^. Compared to other soft actuators, the SMA actuator can produce high force, and compared to electromagnetic motors, it has advantages such as silent actuation, flexibility, and a low profile. Therefore, SMA is suitable as a potential artificial muscle for use in a suit-type soft wearable robot for daily life applications. The large reversible deformation of the SMA is caused by changes in the crystalline arrangement, and it depends on the temperature and state of stress attributed to the native capability of the SMA. The SMA actuator can be stimulated via Joule heating, as shown in Fig. 3d.

The anchor point on the waist was used to hold the wire so that it did not fall off or slip during actuation. The actuation displacement occurred behind the Achilles tendon. The strap on the heel was pulled by the actuator, and the SMA wire’s contracting force acted in parallel with the soleus muscle, which generated a driving torque on the ankle joint. The torque induced the plantar flexion of the ankle, as shown in Fig. 3e.

The electric power conditions for actuation were 71.0 W (1.6 A and 44.4 V) in all experiments. Notably, exposed electrical wires and/or SMA actuators can cause injury such as electric shock and skin burn to the user; however, our actuator was intrinsically safe and unable to cause these injuries because the actuation time was short (approximately 200 ms), which means the applied heat energy is very small, and the encapsulated structure with the Teflon tube insulates the heat of the SMA wire. Therefore, the user is guaranteed safety from skin burns even after cyclic actuation.

The biomechanical analysis in previous work^26^ indicated that the required actuation frequency for walking assistance was approximately 1 Hz. We conducted a study on design parameters (diameter of SMA, heating time, and cooling time) to satisfy the actuation speed of 1 Hz. The user’s intention can be detected by feedback information from sensors attached on the user’s feet, which includes a force-sensitive resistor (FSR), inertial measurement unit (IMU), and electromyography (EMG) sensor.

### Garment pressure measurement

The following experiments were conducted with participants: garment pressure measurement and satisfaction survey, which were approved by the institutional review board (IRB) at Konkuk University. All methods, including experimental protocols, were carried out in accordance with relevant guidelines and regulations of the IRB. All participants provided informed consent before the experiment.

**Figure 4:**
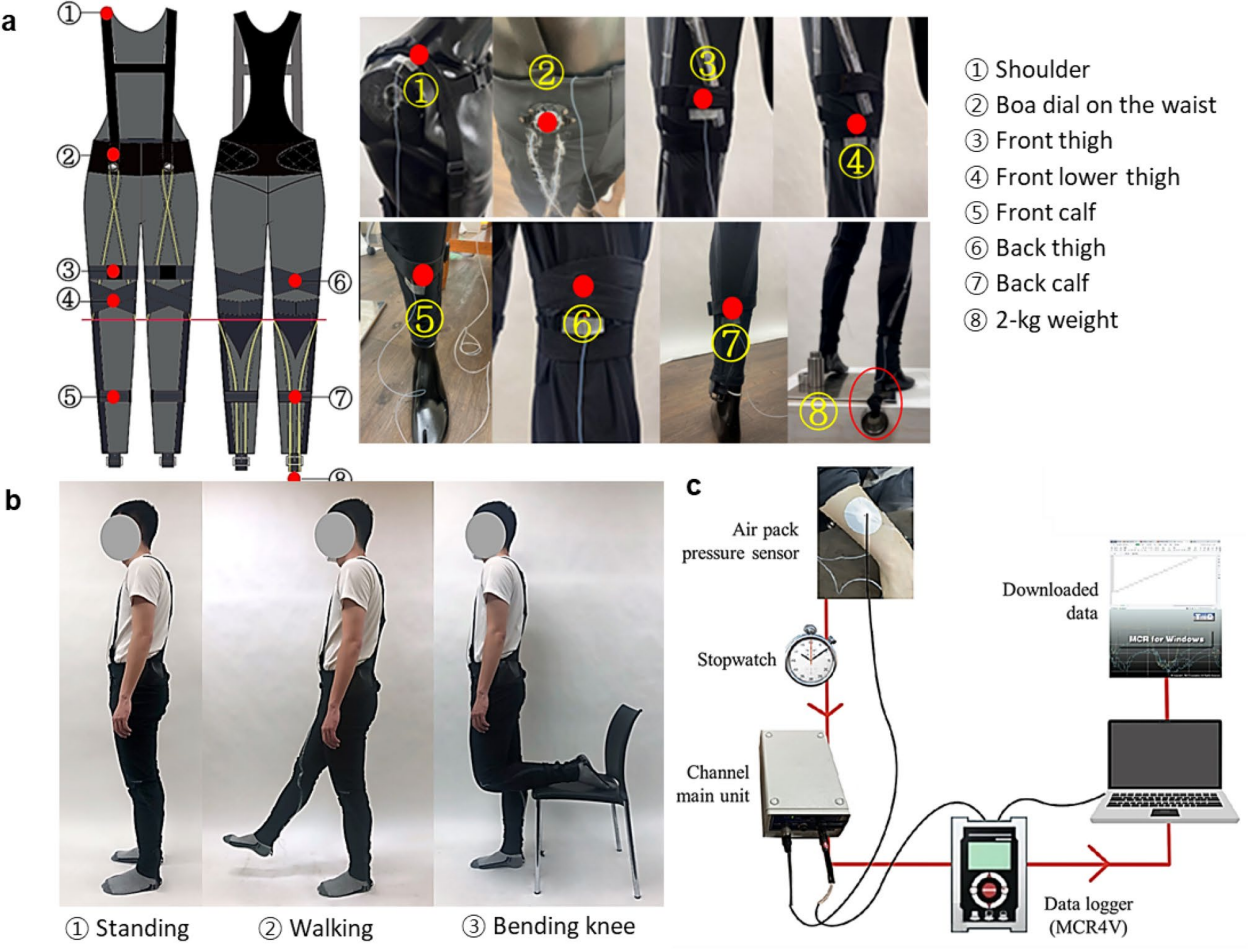
Garment pressure measurement process. (**a**) The garment pressure measuring points, with a total of eight different points. (**b**) Postures for measuring the garment pressure. (**c**) Overview of the garment pressure measuring system.

The garment pressures of the first and second designs were measured while the participants wore them. Furthermore, the differences in the designs with the presence and absence of a harness were compared. A weight of 2 kg was added, and the suit was tested to simulate the actuator performance on the ankle. Compression area, posture change, and wearing duration can affect garment pressure^14–17^, and therefore, these factors were considered when measuring pressure. The pressures were measured at the shoulders, waist, front and back thighs, front lower thigh, and front and back calves^27,28^ (Fig. 4a) when the participants stood, walked, and bent their knees^29,30^, as shown in Fig. 4b. The second design was changed in comparison to the first design, and stitches were added; the front lower thigh was only measured for the first design.

The existing research on measuring garment pressure showed that researchers had experimented with different ranges of height and weight with one garment (within the normal body mass index (BMI) range). Thus, we referred to existing research methods and checked if they were in the normal range of BMI. After our convenience sampling, male volunteers in their 20 s and 30 s with an average BMI participated in the experiment. The mean height, weight, and BMI of the 12 participants were 174.3 cm (standard deviation (SD) = 4.24 and range (R) = 171–182), 68.12 kg (SD = 4.48, R = 60–73), and 22.65, respectively, which were in the normal range of 19–24. We conducted these measurements at least 2 h after the participants had eaten^30^.

The participants were asked to adjust the anchoring parts so that the parts felt simultaneously comfortable and supportive as they would in real life. The researchers also checked if the anchoring parts fit and were sufficiently tight for appropriate functioning, if the important parts and SMA wire lengths were placed next to the target body location, and if the prototype was worn properly. The garment pressure was measured using the air-pack method equipment (AMI 3037-2-2B, Sanko Tsusho), as shown in Fig. 4c. Each participant wore a short-sleeved T-shirt (100% cotton) so that the amount of clothing on the upper body was the same for all participants. Then, measurements were started after checking the stability of the participants while standing or sitting for 30 min^29^. The test environmental conditions were a temperature of 25 ± 1℃, humidity of 65 ± 10% RH, and airflow of 0.5 m/s^29^, and points were marked at the measurement locations and attached sensors when measuring. The garment pressures were measured 2–3 times for each movement, and participants had 10-min rests between each posture^30^. Each part was measured for a total of 60 s. Based on previous research^31^, 10 s were eliminated from the beginning and from the end of the collected data for noise removal, such that the average value for a duration of 40 s was analyzed.

### Satisfaction evaluation

A face-to-face interview survey was conducted with the 12 participants^26^. A total of 43 questions were asked, including 9 demographic questions (gender, age, height, weight, residence area, occupation, health status, garment-wearing habits) and 28 questions about subjective garment pressure (shoulder, waist, front thigh, back thigh, front lower thigh, front calf, back calf, 2-kg weight)^30,31^. The 16 questions on design satisfaction consisted of questions on durability, functionality, design, materials, and purchase and use intentions^26,33–35^ and were based on a 5-point Likert scale, but also included an open-ended question for suggestions. The Cronbach’s alpha value was 0.99 and showed high reliability (> 0.80), and internal consistency was confirmed. Descriptive analyses, such as frequency and percentage, and *t* tests were performed using SPSS 25.0.

### Performance test of the wearable robot

**Figure 5:**
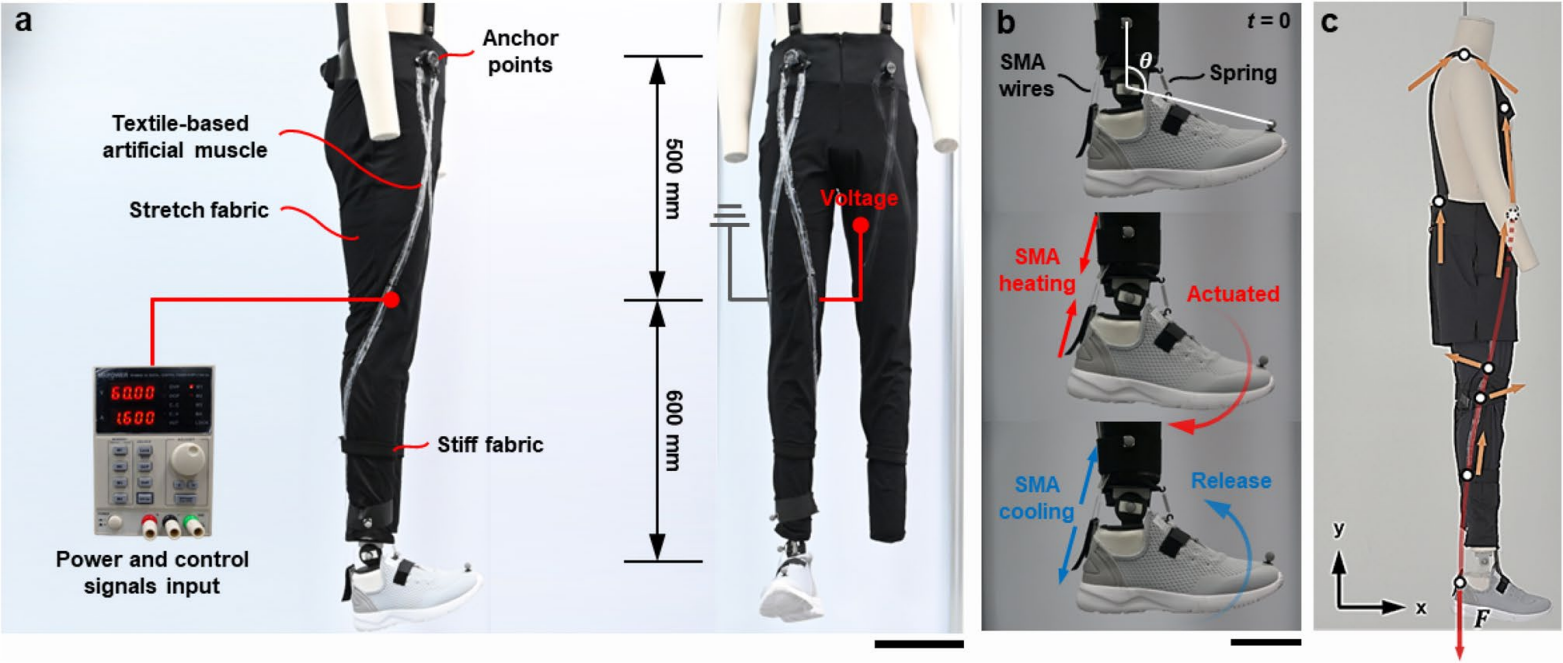
Testing setup. (**a**) Overview of the testing setup for cycling actuation and performance testing of the wearable robot. The scale bar in (**a**) represents 200 mm. (**b**) The sagittal plane ankle angle was measured by the motion capture system. The scale bar in (**b**) represents 100 mm. (**c**) Distributed forces on the garment caused by actuating force.

This experiment was performed with an ankle-articulated mannequin wearing our robots, as shown in Fig. 5a, to evaluate the robot performance. In this experiment, the ankle range of motion and actuation control performance were evaluated. For this purpose, wire-type SMA actuators with a 0.006-in diameter were embedded to generate an assistive force for ankle motion. Power and signal input were controlled by a microcontroller unit (Uno R3; Arduino). The actuators were powered by the input current of the power supply, and ON–OFF switch control was performed by the controller unit and metal–oxide–semiconductor field-effect transistor (MOSFET) logic circuit. SMA actuators 600 mm long were wired at both ends, taking into consideration the 4% strain of the wire-type SMA (Flexinol® actuator wire; Dynalloy).

Commercially available SMA wire (Flexinol® by Dynalloy, Inc.) was used in all experiments. The strain of the SMA wire is typically 3–5% of the i–s original length. In addition, we studied the mechanical and electrical characteristics of the SMA actuator from previous work^26^ and found the stable strain range of the SMA wire. The length of the SMA wire can be adjusted using the Boa dial, depending on the user, and therefore, the desired stress and force to a soleus muscle can be fit according to the user. The actuation stroke of the SMA wire is determined by its original length. Based on the analysis of ankle motions during normal walking^26^, we designed the SMA wire to have an actuation displacement of 2–3 cm, which is 60 cm by wiring, near the knee joint. A variation in the actuation stroke from the inherent differences in height and/or body proportion may cause a slight difference in the actuation force and stroke; however, it can be compensated for by compliance with the SMA actuator. If there is a large dimensional difference in users, the actuation stroke can be tailored by changing the wired points.

In the cyclic actuation test, a spring that could generate an antagonistic force was installed to recover the angle of the ankle after actuation, as shown in Fig. 5b. We measured the angular change over time while applying electrical power input to the SMA wire after adjusting the position of the foot parallel to the ground. The sagittal plane ankle angle was measured using the positions of the three reflectors tracked by the motion capture system (Prime^x^ 13; Optitrack).

The forces are represented by red and orange arrows in Fig. 5c. Red indicates the force direction from the additional mass near the distal ankle joint, and orange indicates the reaction forces near the anchor. Light red lines depict the routing path of the shape-memory alloy actuator. Finally, the white circles indicate anchor points at the shoulder, waist, thigh, and ankle joint. The SMA wire connected from the ankle to the waist generates a contraction force when it is actuated via Joule heating, which results in tugging on the garment. Body parts with larger bones, such as the shoulder, hip and plantar aspects of bilateral feet^36^, support most of the carrying mass and body. The actuating force from the SMA actuator is supported by the anchored waist, as shown in Fig. 5c. The waist textile and shoulder strap (harness) are physically connected by hooks, which consequently supports the substantial loads at the shoulder. Therefore, forces generated by the SMA wire for the ankle joint are distributed throughout the anchor on the entire body, which helps prevent stress and slippage on the local areas of the body during actuation. Based on this analysis, we measured the garment pressure on the various anchor points and body parts (white dots in Fig. 5c) by suspending 2 kg of constant load (corresponding to the maximum actuation force) on the distal ankle. Pressures on the various anchor points and body parts increase (80% increment at the shoulder and waist, which are the main supporting body parts). These experiments indicate that actuation forces were properly distributed across the entire body by our routing design, and a meaningful correlation was found between the actuation force and the measured garment pressure.

The weight for the garment pressure measurement was determined by the force generated by the SMA. Based on the results in previous work^26^, the maximum actuating force was set to 21 N. Therefore, the experiment was conducted by suspending a weight of 2 kg at the distal ankle joint to identify the correlation between garment pressure and actuating forces. In this experiment, SMA wires with a diameter of 0.006 inches were used to assist walking. The diameter of the SMA is a key design parameter that determines various aspects of actuator performance, such as actuation force and frequency. An SMA with a larger diameter can provide a high actuation force, but it has a low actuation frequency. The SMA wire’s smaller diameter provides a low actuation force but has a high frequency. We adopted the SMA with a 0.006-inch diameter to achieve a frequency sufficient for the walking gait, which was suitable for fast actuation at approximately 1 Hz of the walking cycle.

## Results

### First design: garment pressure measurement

**Figure 6:**
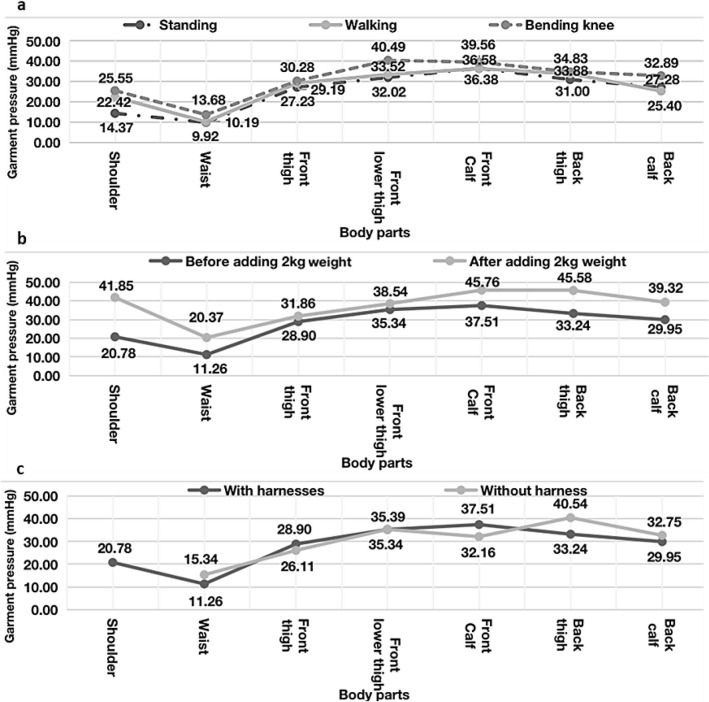
Garment pressure. (**a**) Results of the measured garment pressure and satisfaction-measured garment pressure by posture. (**b**) Measured garment pressure before and after the 2-kg weight was added. (**c**) Garment pressure with and without the harness.

The garment pressures were lowest when participants were standing, increased when participants were walking, and were highest when the participants bent their knees, as shown in Fig. 6a. There were more garment shape changes when participants walked or bent over than when they were standing. The garment pressures were the highest at the front thigh (40.49 mmHg), front calf, and back thigh when participants bent their knees.

On average, the front calf had the highest pressure (37.51 mmHg), followed by the front lower thigh, back thigh, back calf, front thigh, shoulder, and waist. The garment pressure at all measured body areas increased when the 2-kg weight was added to simulate the actuator’s activation and to pull on the pants, as seen in Fig. 6b. The pressure at the shoulder increased the most (101.40%), followed by the waist (80.91%), back thigh (37.12%), back calf (31.29%), front calf (21.99%), front thigh (10.24%), and front lower thigh (9.05%). After the removal of the harness, the garment pressure increased at the waist (+ 36.23%), back thigh (+ 21.96%), and back calf (+ 9.35%); however, the garment pressure decreased at the front thigh (− 9.65%), front lower thigh (− 0.14%), and front calf (− 14.26%), as shown in Fig. 6c.

### First design: satisfaction evaluation

**Figure 7:**
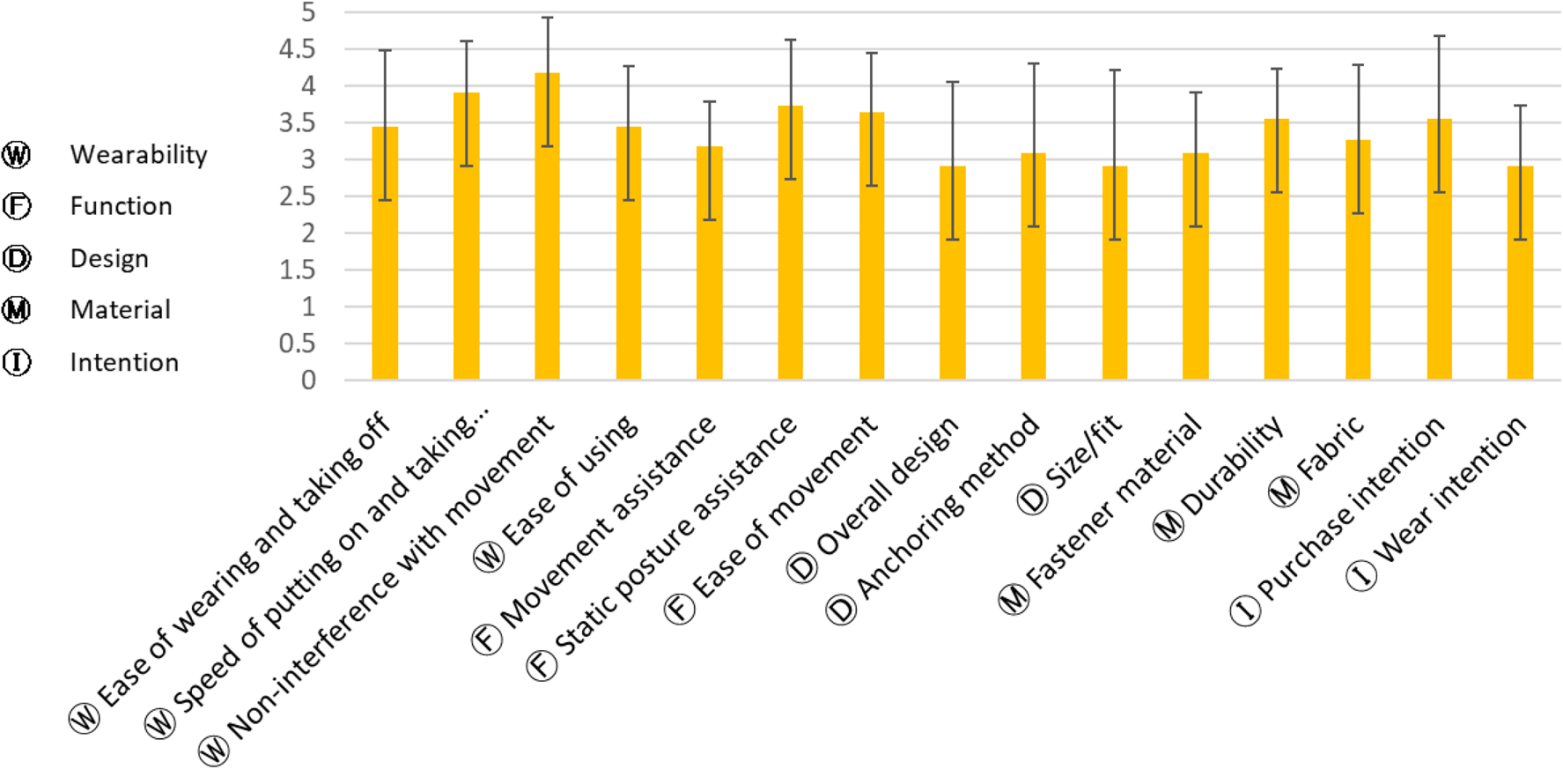
First design: satisfaction evaluation.

Participants evaluated their satisfaction with the first design in terms of wearability, function, design, material, and purchase intentions (Fig. 7). Overall, the participants were satisfied with the first design except for the overall design and size/fit (M = 2.91 for each). The most satisfactory aspect was wearability with respect to noninterference with movement (M = 4.18, SD = 0.75), followed by static posture assistive function and durability. In the design, the anchoring methods were the most satisfactory aspects, and the materials were also satisfactory (M = 3.09–3.55). In the open-ended question, participants wanted to increase the crotch length and to add layers to the crotch parts so they could wear the garment as daily wear. Furthermore, the purchase intention was higher than the wear intention, which may be because of dissatisfaction with the overall design, size/fit, and garment pressure. For the second design, the participants suggested an increase in crotch length and modified two-in-one pants.

### Second design: garment pressure measurement

The second design and prototype were developed based on the results for the previous design (Fig. 2). First, all measured garment pressures for the second design were lower than those for the first design. The area with the greatest decrease was the back thigh (33.24 → 13.55 mmHg; − 59.24%), followed by the front calf (37.51 → 17.07 mmHg; − 54.49%), front thigh (28.90 → 17.63 mmHg; − 39.00%), shoulder (20.78 → 12.76 mmHg; − 38.59%), back calf (29.95 → 23.79 mmHg; − 20.57%), and waist (11.26 → 11.16 mmHg; − 0.89%). These decreases indicate that the design changes decreased the garment pressure (0.89–59.24%) and that the garment pressures for all areas were lowered; no part showed an increased level of garment pressure.

**Figure 8:**
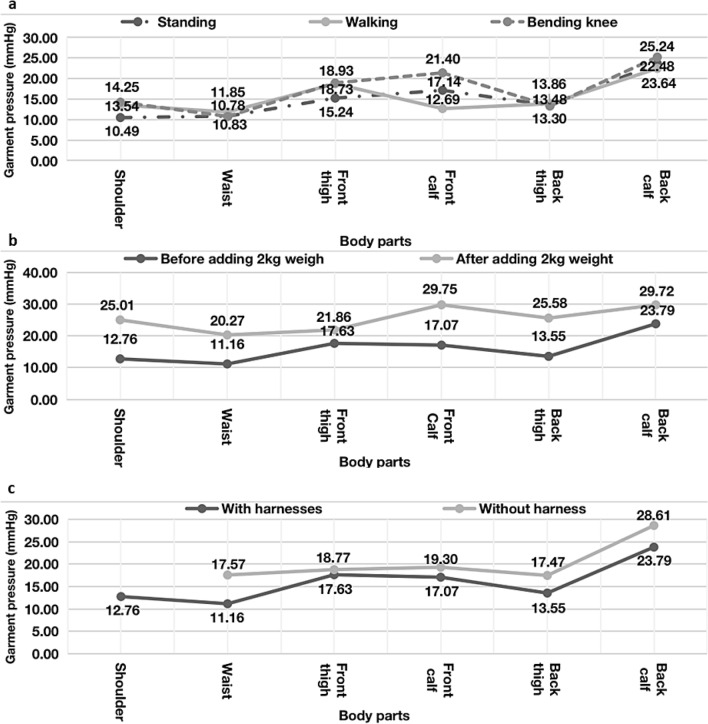
Results of measured garment pressure and satisfaction. (**a**) Measured garment pressure by posture. (**b**) Measured garment pressure before and after the 2-kg weight was added. (**c**). Garment pressure with and without the harness.

Second, garment pressures for the second design ranged from 20.27 to 29.75 mmHg when weight was added (Fig. 8b). These values were lower than those for the first design (20.37–45.76 mmHg). The shoulder showed the greatest increase (48.98%), followed by the back thigh (47.03%), waist (44.94%), front calf (42.62%), back calf (19.95%), and front thigh (19.35%).

Third, the harness of the second design decreased the garment pressure for all measured body areas (Fig. 8c). The greatest changes after the removal of the harness were at the waist (+ 57.44%), back thigh (+ 28.93%), back calf (20.26%), front calf (13.06%), and front thigh (6.47%).

### Second design: satisfaction evaluation

**Figure 9:**
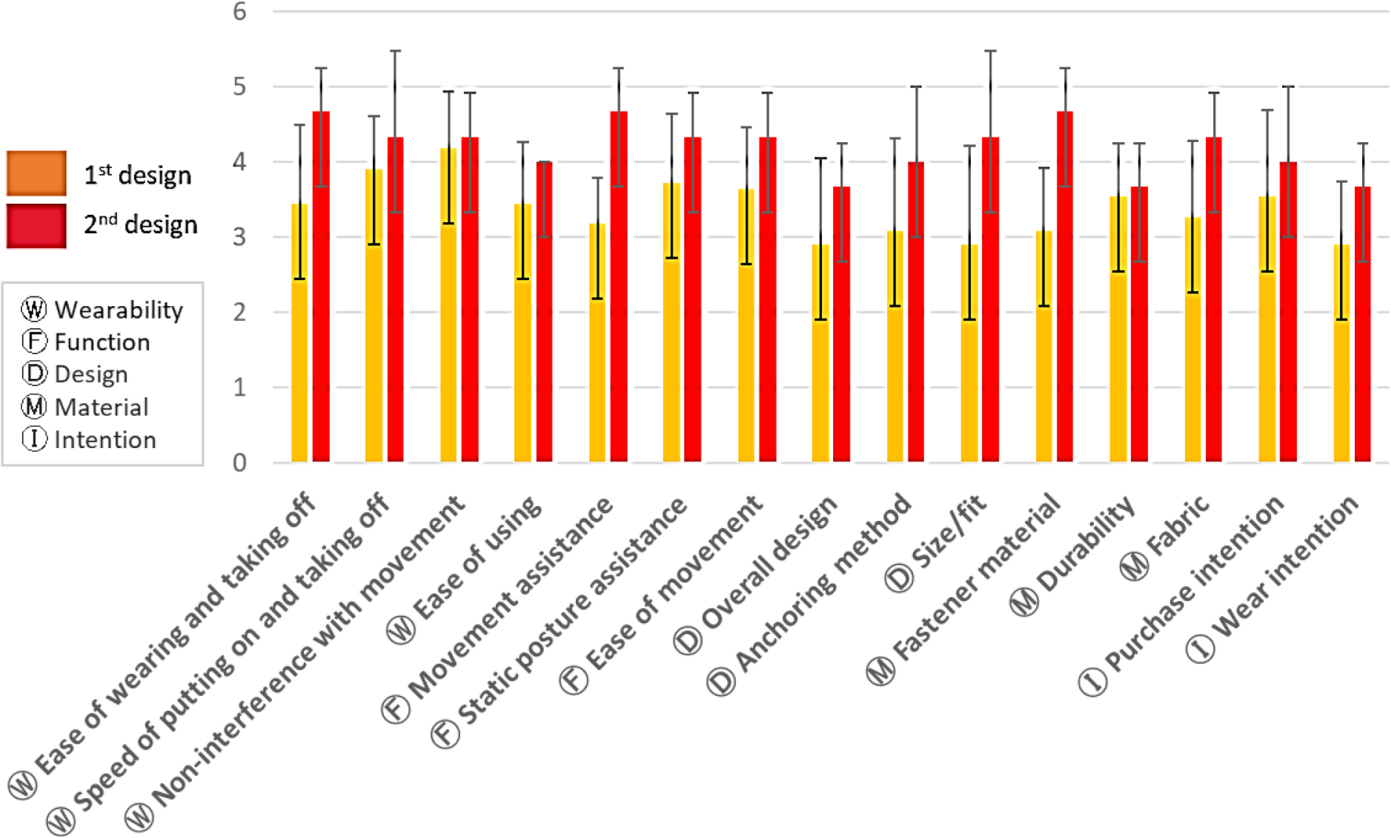
Differences in satisfaction between the first and second designs.

A comparison of the levels of satisfaction between the first and second designs revealed that satisfaction increased with regard to all aspects for the second design (Fig. 9). After the *t* test, the second design showed a significantly increased level of satisfaction with wearability (t =  − 3.225, *p* < 0.01), function (t =  − 7.147, *p* < 0.001), design (t =  − 3.830, *p* < 0.01), and material (t =  − 3.601, *p* < 0.001) compared to these measures for the first design. The unsatisfactory aspects of the first design, such as overall design and size/fit, were improved to satisfactory for the second design. Both purchase and wear intentions increased for the second design compared to those for the first design (t =  − 3.225, *p* < 0.001).

### Performance test results

**Figure 10:**
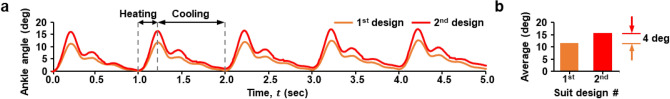
Results of the cyclic actuation and performance tests for the suit-type soft wearable robots. (**a**) Cyclic actuation with an electric input of 1.6 A for the first and second designs. (**b**) Average rotation angles of the first and second designs.

Figure 10a shows the result of cyclic actuation with an electric input of 1.6 A for both suit designs. A single actuation cycle has a heating time of 200 ms and a cooling time of 800 ms. In this experiment with the testbed (Fig. 10b), the maximum rotation angles are 11.8° in the first design and 15.8° in the second design.

## Discussion

### First design: garment pressure measurement and satisfaction evaluation

First, the waist was at the suggested level of approximately or less than 20 mmHg^14,15^; however, the other areas were higher than that. The shoulder was under comfort garment pressure for a few scenarios, such as for bending the knee before adding the 2-kg weight and with a harness. We recommend modifying the first design to lower the garment pressure, especially at the front calf and the lower front and back thighs, which had garment pressures higher than the comfort range of 20–30 mmHg^14^.

Second, because the shoulder area holds the weight, it is necessary to have shoulder components. It is advisable to decrease the garment pressure, especially on the front calf, back thigh, and shoulder. The front lower thigh showed a low garment pressure because the fixing force of the belt decreased because of the added weight.

Third, the magnitude of the increases in pressure was greater than that of the decreases in pressure. Thus, the harness helped decrease the garment pressure at the waist, back thigh, and calf. This may be because the actuator was on the back calf area. This can help decrease the garment pressure on the back side of the suits. However, it is also advisable to decrease the garment pressure in the front areas.

### Design modifications: second design and prototype

First, the front calf and the front and back thighs showed the top three highest garment pressures, with the X-shaped anchoring band being fixed in the pants seams. Second, the shoulder showed the greatest increase in garment pressure when weight was added, followed by the waist. Therefore, we modified the shoulder and waist areas by changing the waist band fabric and adding a wider and rounded harness to better support the shoulders. Third, in the open-ended questions, participants suggested two-in-one pants to cover the fitted leggings so they could be worn on a daily basis. Thus, a short pants design was added in the second design for a better design. Fourth, the Teflon tube started from the Boa dials and passed through the connection hole of the shorts, and it was fixed to the surface of the long pants. Notably, the equipment that weighs the most, such as batteries and drivers, requires the wearer to have a relatively large surface area to relieve pressure. Therefore, the pocket for inserting electronic components, such as batteries, was located between the waist and the hip to relieve weight pressure without restricting movement^37^.

### Second design: garment pressure measurement and satisfaction evaluation

In this design, we focused primarily on modifying areas other than the waist because the waist had an acceptable pressure level in the first design; thus, the waist showed the least change in the second design. All garment pressures (11.16–23.79 mmHg) were lower than the range of the comfort zone (20–30 mmHg), with the back calf (23.79 mmHg) having the highest pressure, and where the actuator was placed with high stability (Fig. 8a). Thus, the second design showed an improvement over the first design with respect to garment pressure, and it could be further modified to lower the back calf garment pressure while maintaining the stability of actuators in a future design.

For the first design, the garment pressure decreased in the shoulder and waist but increased at the front thigh, front calf, and back thigh. Thus, compared to that in the first design, the garment pressure on the shoulder and waist areas in the second design was effectively distributed to the front thigh, front calf, and back thighs. In contrast, in the first design the harness increased the garment pressures on the waist, back thigh, and back calf. This supports the conclusion that the harness of the second design helped decrease the garment pressure at the waist, back thigh, and back calf. It is thus advisable to employ a harness to distribute the garment pressure for the suit-type soft wearable robot platform. Overall, the satisfaction with the second design was higher than that with the first design, including purchase intention.

### Performance test results

The results for the first design indicated that a high actuation force generated by the SMA caused the deformation of the garment to create slippage. Thus, the anchoring parts on the waist and heel were subsequently used to reduce slippage during actuation. The performance of the second design was improved by using stiffer band fabrics (Fabric E) and increasing the width of the 3D-printed waist support to reduce the deformation of the garment.

### Design guidelines

The following design guidelines are suggested. First, it is better to design garments to be like those worn in daily life, such as in the style of two-in-one pants. Second, for movement comfort and a better fit, it is recommended to make size-adjustable components such as Boa dials, buckles and slides, hooks and loops, webbing bands, stretchy fabrics, and elastic bands. Third, to make the garment more comfortable to wear while moving around, side slits can be added to the pants, and pocket pouches with electrical circuits can be placed on the waist back. Fourth, a wider and rounder harness on the shoulder can enhance stability and comfort. Fifth, to reinforce the strength and durability, a two-layered harness and a waist band can be used. Next, for ease of wearing and taking off, a front concealed zipper is suggested. Finally, it is recommended to use stretchy fabric overall, with mesh fabric for zones that are prone to sweatiness, and to use non-stretchy fabrics for the front waist and harness to withstand pulling power.

## Conclusion

In this study, two versions of suit-type walking-assistive wearable robot platforms were developed, and garment pressure and satisfaction after wearing were evaluated. The second design decreased the garment pressure and increased user satisfaction, and it was therefore recommended for the suit-type platform.

1. The garment pressures for the second design were all lower than those for the first design. The garment pressure when standing was the lowest, and the overall garment pressures increased progressively when participants walked and bent their knees. This shows that movements with more extreme angles increase garment pressure.
2. When weight was added, the garment pressure increased less in the second design and remained lower than that in the first design. The garment pressures applied to the shoulder and waist areas were better distributed to the front thigh, front calf, and back thighs in the second design than in the first design.
3. The garment pressure of the second design increased for all measured areas when the harness was removed. The greatest changes were at the waist, back thigh, and back calf. It is therefore suggested to use a harness to distribute the garment pressure for the suit-type soft wearable robot.
4. User satisfaction increased greatly for the second design compared to that for the first design (0.01 < *p* < 0.001). The function increased the most, the previously unsatisfactory aspects of overall design and size/fit became satisfactory, and the intentions concerning purchase and wear improved for the second design compared to those for the first design.
5. Finally, the second design showed larger maximum rotation angles than those of the first design. The high actuation force generated by the SMA caused the deformation of the garment to create slippage, while the anchoring components of the second design reduced slippage during actuation.

This research provides strategies for designing suit-type walking-assistive wearable robot platforms to provide comfortable garment pressure and to satisfy wearers. The modified second design showed decreased garment pressure and higher satisfaction than the first design. We hope that the developed designs and guidelines will help in the development of wearable robots and that continuous modification will have a positive effect on the commercialization of wearable robots by increasing wearer satisfaction.

All garment pressures were lower than the suggested level for comfort except at the back calf, where the actuator is located. Future studies can focus on how to decrease the garment pressure on the actuator locations as well. It is recommended different sizes of suits be developed for diverse body sizes to see if there are any differences in results with size-adjustable suits. Furthermore, different types of suits should be designed with different actuator types for diverse users to fit adequately and to accommodate different needs related to gender, age, and targeted body areas.

This study is expected to help in the development of comfortable-to-wear and satisfactory suit-type soft wearable robots by providing design strategies and development processes for wearable robot developers.

## Data Availability

The datasets used and/or analyzed during the current study are available from the co-corresponding authors on reasonable request.
